# Direct Comparative Safety and Anatomical Performance of Amulet Versus Watchman Devices for Left Atrial Appendage Occlusion

**DOI:** 10.7759/cureus.93019

**Published:** 2025-09-23

**Authors:** Glen Silva Rojas, Kevin J Silva-Rojas, Ariana N Zea Olvera, Galo G Farfan Cano

**Affiliations:** 1 Medicine, Catholic University of Santiago of Guayaquil, Guayaquil, ECU; 2 Health Sciences, Catholic University of Santiago of Guayaquil, Guayaquil, ECU; 3 Research, FarCan, SilRoj et al. Research Group, Guayaquil, ECU

**Keywords:** left atrial appendage closure (laac), left atrial appendage (laa), randomized controlled trial (rct), review article, systematic review

## Abstract

Atrial fibrillation (AF) is associated with increased risk of cardioembolic events, particularly ischemic stroke, and left atrial appendage (LAA) occlusion devices have emerged as alternatives to long-term anticoagulation in high-risk patients. This systematic review evaluates the comparative safety and anatomical performance of the Amplatzer Amulet (Abbott, Chicago, IL, US) and Watchman (Boston Scientific, Marlborough, MA, US) devices based on two head-to-head randomized controlled trials (RCTs), Amulet IDE (investigational device exemption) and SWISS-APERO, which provided complete follow-up data on clinical and procedural outcomes. Stroke incidence, major bleeding, and cardiovascular or all-cause mortality were similar between devices, reflecting comparable safety profiles. However, anatomical outcomes differed, with the dual-seal design of Amulet achieving lower rates of peri-device leaks, suggesting a potential structural advantage. Complementary studies addressing procedural techniques, arrhythmia recurrence, and post hoc mechanistic analyses were discussed to contextualize these findings, though they were excluded from primary quantitative synthesis due to heterogeneity in endpoints and follow-up. Methodological limitations, including small sample sizes, variable outcome definitions, and reliance on the Jadad scale for risk-of-bias assessment, constrain the generalizability of results. Despite these limitations, this review provides an updated synthesis of contemporary evidence on LAA closure, highlighting that both devices are effective in reducing thromboembolic risk while emphasizing the importance of procedural strategy, operator experience, and device-specific anatomical considerations in optimizing outcomes.

## Introduction and background

Atrial fibrillation (AF) is the most common sustained cardiac arrhythmia worldwide, characterized by atrial electrical asynchrony with ectopic impulses predominantly arising from the pulmonary veins [1,2]. It is associated with a 3%-6% annual mortality rate, which increases substantially in the presence of complications such as cerebral thromboembolism, particularly among patients with recurrent or treatment-resistant arrhythmias. Catheter ablation has emerged as a cornerstone intervention aimed at restoring sinus rhythm [3,4]; however, its long-term efficacy is limited by an approximate 45% recurrence rate, with nearly half of these recurrences occurring during the post-procedural blanking period-a three-month window in which arrhythmias may reappear without constituting procedural failure [5].

In non-valvular AF (NVAF), up to 90% of thrombi originate in the left atrial appendage (LAA), making it the principal source of cardioembolic events. Standard management typically combines arrhythmia control through catheter ablation with long-term anticoagulation to mitigate embolic risk. The advent of percutaneous LAA closure devices, such as Watchman™ (Boston Scientific, Marlborough, MA, US) and Amplatzer Amulet™ (Abbott, Chicago, IL, US), offers an alternative thromboprophylactic strategy by physically occluding the LAA [6-9]. These devices have demonstrated reductions of up to 70% in ischemic event incidence according to CHA₂DS₂-VASc scores [6,10,11], yet their long-term safety and comparative efficacy remain under evaluation in ongoing trials, including CHAMPION-AF and CATALYST [2,12]. Given the expanding clinical adoption of LAA closure in patients with recurrent AF, contraindications to oral anticoagulation, and high thromboembolic risk (CHA₂DS₂-VASc ≥ 2 in men, ≥3 in women), a systematic synthesis of available evidence is warranted [12-17]. This review critically evaluates the effectiveness, safety, and potential of these devices as long-term alternatives to indefinite anticoagulation therapy.

## Review

Materials and methods

This systematic review was conducted in accordance with the Preferred Reporting Items for Systematic Reviews and Meta-Analyses (PRISMA 2020) guidelines [18].

Search Strategy

The search strategy used was (("Left Atrial Appendage Closure") OR ("Atrial Appendage")) AND (randomized controlled trial OR RCT).

Filters Included

The filters applied included studies published within the last five years, free full-text availability, and randomized controlled trial (RCT) design.

Eligibility and Exclusion Criteria

The eligibility criteria include studies meeting all of the following: (1) published within the last five years, (2) free full text, and (3) RCT design. The exclusion criteria include studies involving HIV-positive populations, pediatric or elderly populations (>65 years), pregnant women, letters to the editor, systematic reviews, and meta-analyses.

The review focused on adult patients with NVAF undergoing LAA occlusion (LAAO), aiming to synthesize current evidence regarding Amulet vs. Watchman devices. Populations with significant comorbidities or extreme age groups were excluded to reduce confounding factors that could bias the safety and efficacy outcomes. This approach ensures that included studies provide directly comparable data for the primary endpoints (stroke, cardiovascular mortality, and major bleeding) and allows a focused, methodologically sound synthesis.

Data Extraction and Risk of Bias Assessment

Two independent reviewers extracted data from eligible studies, including sample size, follow-up duration, device type, and primary and secondary outcomes. Discrepancies were resolved by consensus. Study quality was assessed using the Jadad scale [19], with scores ≥ 3 considered methodologically acceptable. The choice of the Jadad scale, rather than Cochrane RoB 2.0, was pragmatic given the small number of RCTs retrieved and the need to prioritize studies with complete follow-up and reliable reporting.

Summary Methods

Given the heterogeneity of interventions, outcomes, and follow-up periods across studies, a qualitative synthesis was performed. Only head-to-head trials comparing Amulet and Watchman devices (Amulet IDE (investigational device exemption) [11] and SWISS-APERO [12]) were included in the primary analysis. Other studies (Peri-device leaks (PDL) Mechanisms [17], aMAZE [1], and ATP vs. conventional TSP [9]) were discussed for contextual insights but excluded from quantitative comparisons. This systematic approach ensures the reproducibility of study selection, data extraction, and synthesis while providing a focused evaluation of current evidence in LAAO.

Study Selection

**Figure 1:**
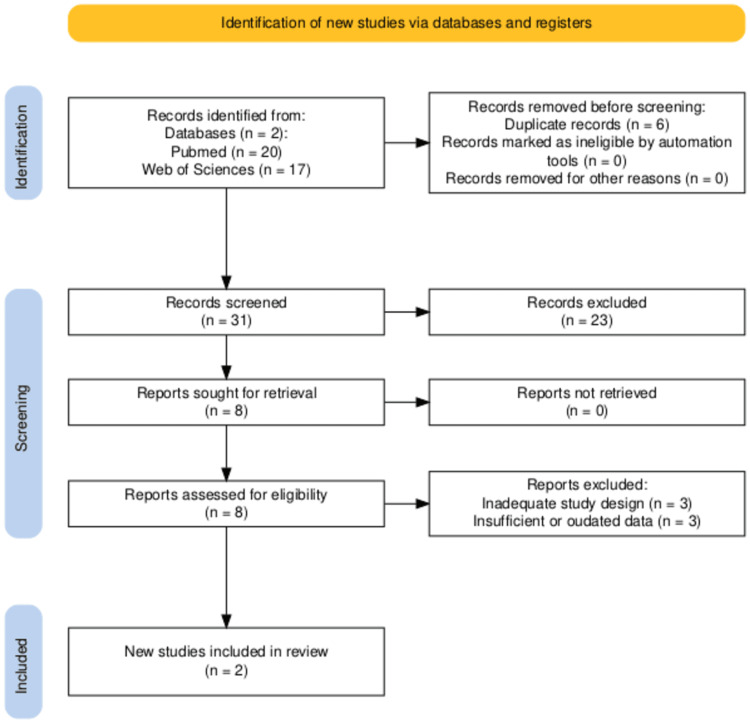
PRISMA flow diagram The figure was created using the PRISMA2020 R package and the Shiny app [20]. PRISMA: Preferred Reporting Items for Systematic Reviews and Meta-Analyses.

A total of 37 records were identified. After removing six duplicates, 31 titles/abstracts were screened. Eight studies met preliminary eligibility for full-text review. Six were excluded due to inadequate design (n = 3), outdated/insufficient data (n = 1), or out-of-scope populations (n = 2). Ultimately, two RCTs (Amulet IDE and SWISS-APERO) were included for analysis. Additional trials (PDL Mechanisms, aMAZE, and ATP vs. TSP) are briefly mentioned for contextual purposes but were excluded from the main synthesis as they did not directly compare Amulet vs. Watchman or did not assess embolic outcomes (Figure 1, elaborated with PRISMA2020 R package and Shiny app [20]).

Results

After applying the predefined inclusion and exclusion criteria, two head-to-head RCTs met all requirements for the primary analysis: Amulet IDE [11] and SWISS-APERO [12] (Table 1). These trials provided complete follow-up data and reported outcomes relevant to safety and efficacy in LAAO. The remaining studies (aMAZE [1], PDL Mechanisms [17], and ATP vs. conventional TSP [9]) were excluded from the primary analysis due to incomplete outcome reporting, differences in study objectives, or insufficient follow-up. They are discussed in the Discussion section to provide context but were not included in quantitative comparisons.

**Table 1:** Characteristics of the included studies IDE: investigational device exemption; G1: Group 1; G2: Group 2; CV: cardiovascular.

Study	Year	Total sample	Analyzed sample	Group 1	Group 2	Stroke G1	Stroke G2	Mortality G1	Mortality G2	CV mortality G1	CV mortality G2	Bleeding G1	Bleeding G2	Jadad
Amulet IDE [11]	2023	1,878	1,833	Amulet (n = 917)	Watchman (n = 916)	4.25	3.39	14.6	17.9	6.6	8.5	16.1	14.7	5
SWISS-APERO [12]	2023	221	221	Amulet (n = 111)	Watchman (n = 110)	Not described	Not described	Not described	Not described	10.0	10.2	7.2	6.9	4

Both trials shared the primary objective of reducing cardioembolic events, primarily ischemic stroke, in patients with NVAF undergoing LAAO. Significant heterogeneity in study design and outcome definitions prevented data pooling for meta-analysis.

In the Amulet IDE trial [11], stroke incidence was slightly higher in the Amulet group (4.25%) compared to Watchman (3.39%), without clinical significance. Cardiovascular mortality was 6.6% for Amulet versus 8.5% for Watchman, and all-cause mortality was 14.6% versus 17.9%, respectively. Major bleeding rates were similar (16.1% vs. 14.7%). The trial achieved a Jadad score of 5, reflecting high methodological quality.

The SWISS-APERO study [12] focused on residual appendage patency and cerebrovascular events. Neurological events were low and comparable (2.7% for Amulet vs. 3.7% for Watchman), as were device-related thrombosis rates (2.4% vs. 3.8%). Major bleeding (7.2% vs. 6.9%) and cardiovascular mortality (10.0% vs. 10.2%) were also similar. This study scored 4 on the Jadad scale.

The three studies excluded from quantitative analysis-PDL Mechanisms [17], aMAZE [1], and ATP vs. conventional TSP [9]-were not comparable due to differences in endpoints, follow-up duration, or procedural focus. They are referenced in the Discussion to provide context for device performance, procedural strategies, and arrhythmia outcomes.

Discussion

This systematic review included five RCTs evaluating LAAO strategies in patients with NVAF, focusing primarily on the Amplatzer Amulet and Watchman devices (versions 2.5 and FLX). For the primary quantitative analysis, only two head-to-head trials (Amulet IDE [11] and SWISS-APERO [12]) were considered, as these provided complete follow-up data relevant to safety and efficacy. The remaining studies (PDL Mechanisms [17], aMAZE [1], and ATP vs. conventional TSP [9]) were not included in the main analysis due to differences in study objectives, incomplete outcome reporting, or insufficient follow-up, but are referenced here for contextual purposes.

Anatomical closure success and the incidence of PDL varied across studies. The Amulet device consistently demonstrated lower rates of severe leaks compared to Watchman, likely reflecting the advantage of its dual-seal design, which combines lobe anchoring with disc coverage [9,10]. However, heterogeneity in PDL definitions and imaging modalities (transesophageal echocardiography (TEE) vs. coronary computed tomography angiography (CCTA)) limits direct comparisons. The PDL Mechanisms trial [17] further highlighted potential structural advantages of the Amulet device, particularly in complex LAA anatomies, although it did not report hard clinical endpoints such as stroke or mortality.

Both the Amulet IDE [11] and SWISS-APERO [12] trials reported comparable rates of major clinical outcomes, including stroke, cardiovascular mortality, and major bleeding. The Amulet IDE trial showed a non-significantly higher incidence of stroke in the Amulet group (4.25% vs. 3.39%) but lower cardiovascular mortality (6.6% vs. 8.5%). SWISS-APERO observed low and similar rates of neurological events, device-related thrombosis, and major bleeding (2.7% vs. 3.7%, 2.4% vs. 3.8%, and 7.2% vs. 6.9%, respectively).

Procedure-related complications were most frequently associated with device positioning and sizing, particularly for the Amulet device, which requires greater operator experience due to its more complex design [17]. Similarly, the ADVANCE-LAAO study [9] suggested that procedural strategies, such as balloon-assisted transseptal puncture, can influence procedural success independently of device type, with higher success rates and lower complication rates observed compared to conventional techniques (92.4% vs. 77.3%; p = 0.001).

The aMAZE trial [1], focusing on arrhythmia recurrence, and the ATP vs. TSP trial [9], assessing procedural techniques, were not directly applicable to embolic outcomes but provide context on the broader procedural landscape. Including these studies in the Discussion ensures transparency and acknowledges available evidence outside the primary head-to-head RCTs.

Overall, the available evidence suggests therapeutic equivalence between Amulet and Watchman devices, with minor anatomical and procedural differences. Heterogeneity in study design, endpoint definitions, follow-up duration, and procedural variables precludes robust meta-analysis and limits conclusions regarding long-term safety and efficacy. Future studies should aim to standardize definitions for procedural success, adverse events, and follow-up criteria while including diverse patient populations and device-specific complications to optimize evidence-based decision-making in LAAO.

Limitations

This systematic review has several important limitations. First, the number of eligible RCTs was small, with only two head-to-head studies (Amulet IDE [11] and SWISS-APERO [12]) fully meeting the inclusion criteria and providing complete follow-up data. The limited sample restricts the generalizability of findings and precludes robust meta-analytic synthesis.

Second, the heterogeneity in study design, endpoint definitions, and follow-up duration across trials prevented data pooling and reduced comparability. Differences in definitions of PDL, imaging modalities, and procedural techniques may have influenced reported outcomes and limited direct comparisons.

Third, risk of bias assessment relied on the Jadad scale rather than the Cochrane RoB 2.0 tool. While the Jadad scale provided a pragmatic method to screen studies for methodological quality, it offers a less comprehensive domain-based evaluation, particularly regarding allocation concealment and selective reporting. Future reviews with a larger number of trials should employ RoB 2.0 for a more robust assessment.

Fourth, most included trials were industry-sponsored, potentially introducing conflicts of interest and selective outcome reporting. Additionally, certain populations-such as elderly patients (>65 years) and those with high comorbidity burden-were systematically excluded, limiting external validity and applicability to real-world clinical settings. Finally, procedural factors such as operator experience, device sizing, and adjunctive techniques (e.g., balloon-assisted transseptal puncture) were variably reported and may have affected outcomes independently of the device type.

Taken together, these limitations underscore the need for cautious interpretation of comparative efficacy and safety between Amulet and Watchman devices. While current evidence suggests similar clinical outcomes, future high-quality, multicenter RCTs with standardized endpoints, longer follow-up, and broader patient representation are necessary to confirm these findings.

## Conclusions

This systematic review consolidates the most robust head-to-head evidence comparing Amulet and Watchman devices for LAAO in patients with NVAF. Both Amplatzer Amulet and Watchman devices demonstrate comparable safety for LAAO, with similar rates of stroke, cardiovascular mortality, and major bleeding. Amulet’s dual-seal design consistently shows lower PDL rates, suggesting an anatomical advantage, though heterogeneity in study designs and endpoints limits definitive comparisons. These findings highlight that device selection should consider anatomical suitability and operator experience. While both devices effectively reduce thromboembolic risk, the observed anatomical differences may influence long-term efficacy and follow-up strategies. Further multicenter studies with standardized endpoints are warranted to confirm these anatomical advantages and optimize patient-specific device choice.
